# Supplementary figures and images for: Potential Role of Photosynthesis in the Regulation of Reactive Oxygen Species and Defence Responses to Blumeria graminis f. sp. tritici in Wheat
Source: Int J Mol Sci. 2020 Aug 11;21(16):5767. doi: 10.3390/ijms21165767 (PMC7460852; doi:10.3390/ijms21165767)

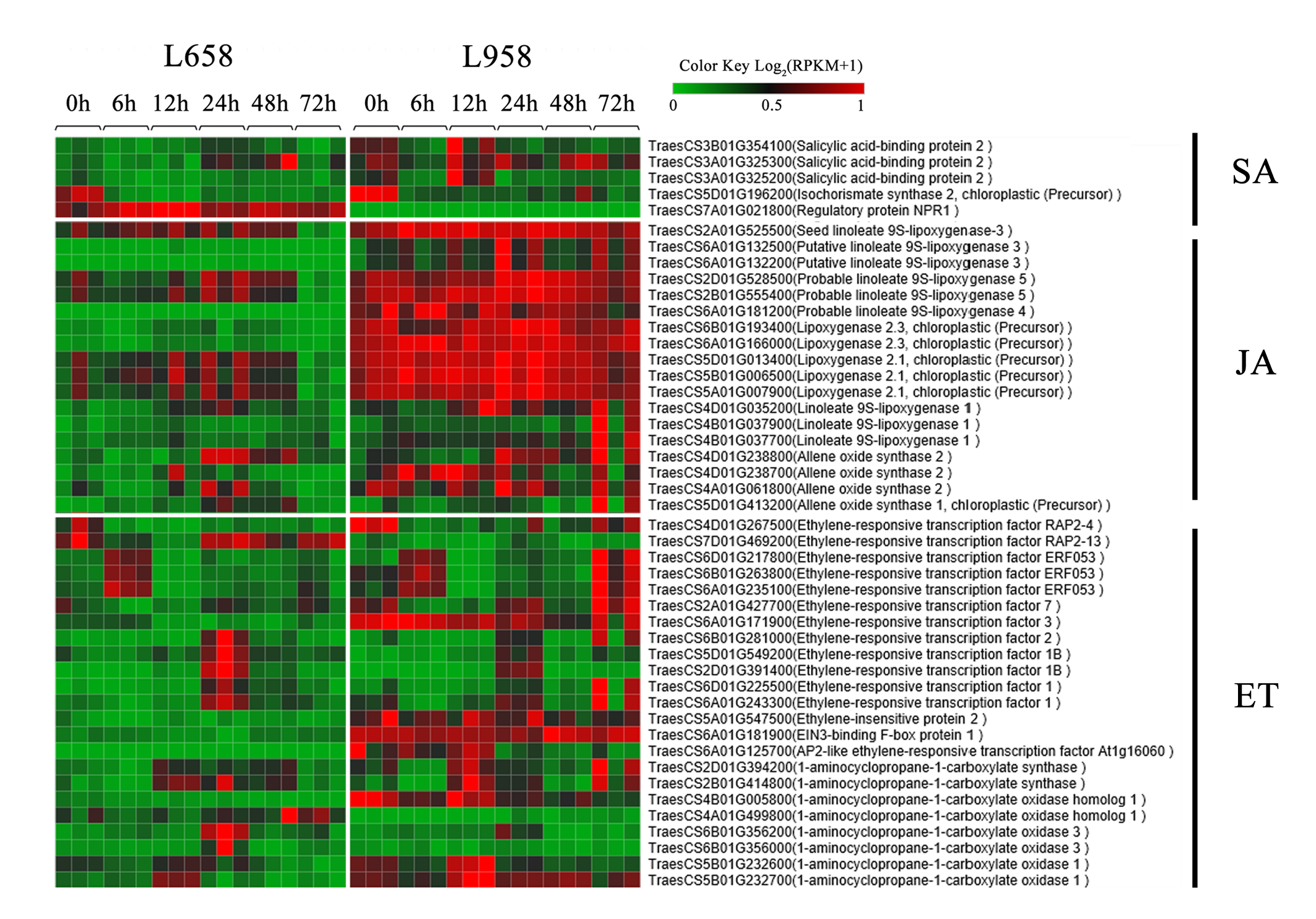

Supplement: Supplementary file 1 [file ijms-21-05767-s001.zip › supplement/Figure S1.tif]

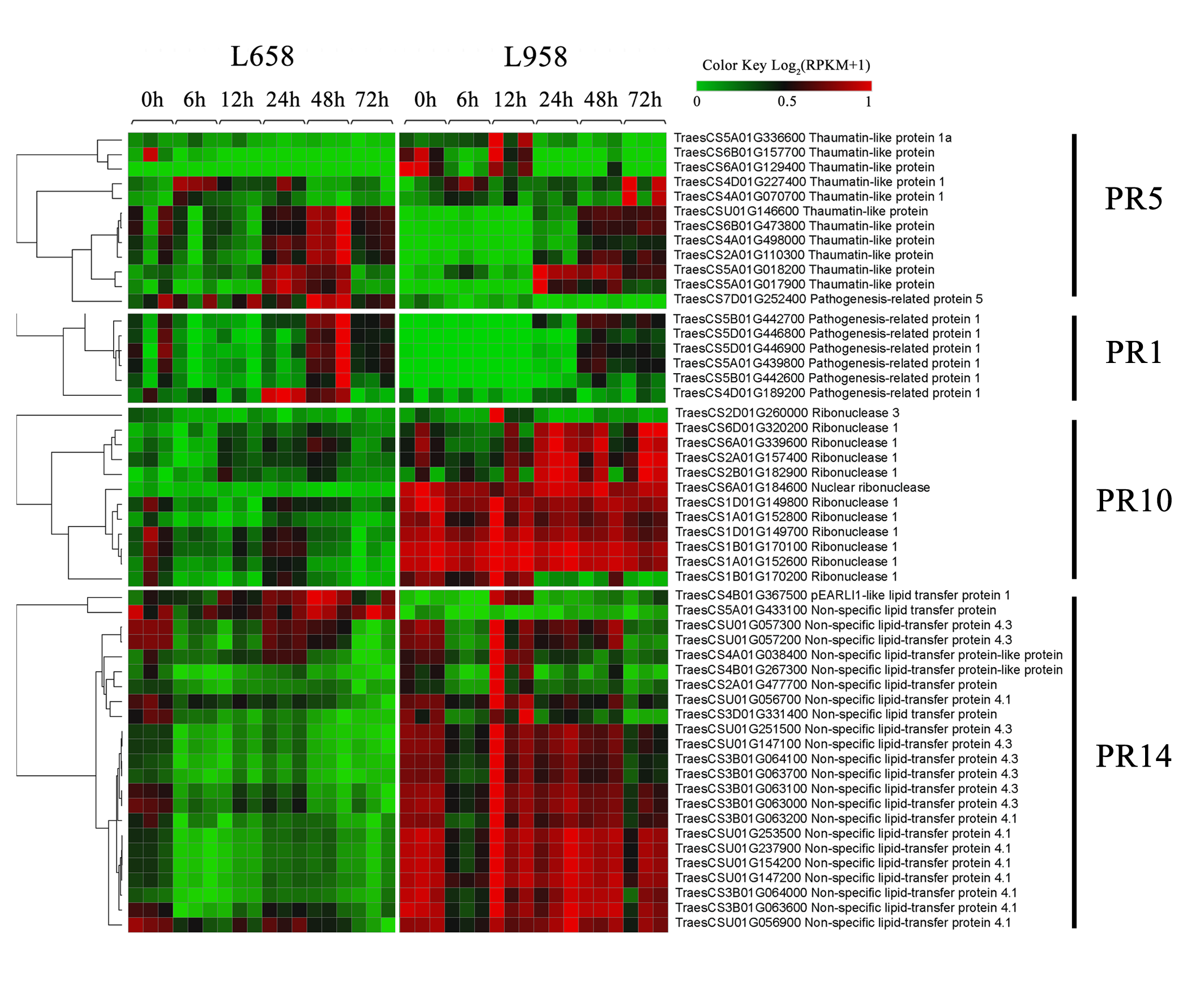

Supplement: Supplementary file 1 [file ijms-21-05767-s001.zip › supplement/Figure S2.tif]

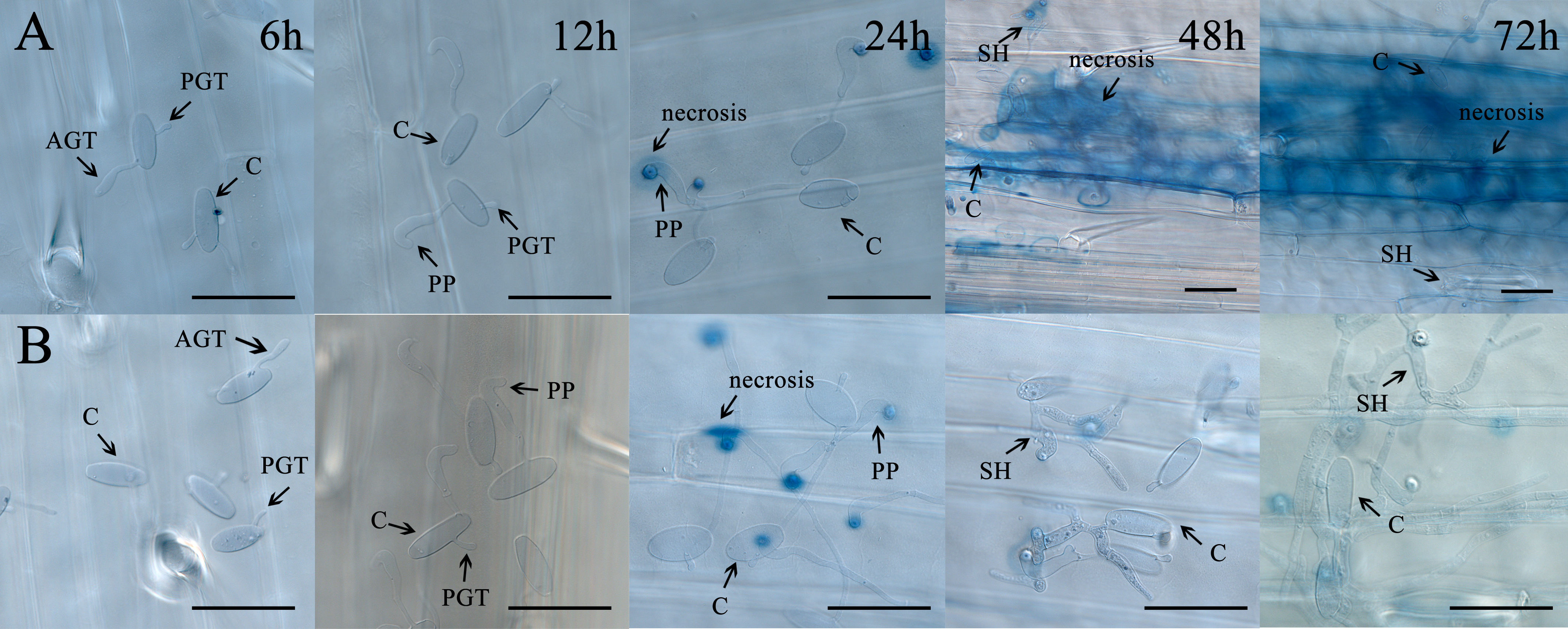

Supplement: Supplementary file 1 [file ijms-21-05767-s001.zip › supplement/Figure S3.tif]
